# Artificial intelligence and extracellular vesicles in oncology: towards tumor diagnosis, prediction, and therapy

**DOI:** 10.1080/10717544.2026.2713415

**Published:** 2026-08-12

**Authors:** Xiangyu Li, Hao Su, Yaping Li, Longyang Jiang, Jie Zhou, Liaoyun Zhang, Yilan Huang, Xuping Yang, Min Li

**Affiliations:** a Department of Pharmacy, The Affiliated Hospital, Southwest Medical University, Luzhou, China; b School of Pharmacy, Southwest Medical University, Luzhou, China; c Department of Pharmacy, Sichuan Provincial Woman’s and Children’s Hospital & The Affiliated Women’s and Children’s Hospital of Chengdu Medical College, Chengdu, Sichuan, China

**Keywords:** Artificial intelligence, extracellular vesicles, tumor diagnosis, tumor prediction, tumor therapy

## Abstract

Extracellular vesicles (EVs) have emerged as promising tools for early cancer detection, therapeutic monitoring, and drug delivery in oncology. Artificial intelligence (AI), particularly machine learning and deep learning, offers new analytical tools and computational approaches for EV research. This review summarizes recent advances in the application of AI to EV isolation, characterization, diagnosis, and drug delivery, with particular emphasis on its potential to enhance tumor detection sensitivity, diagnostic accuracy, and the rational design of delivery platforms. Special attention is given to the roles and recent applications of AI models in integrating multimodal features, characterizing EV heterogeneity, supporting diagnostic classification, and modeling in vivo behavior. Moreover, we examine the integration of AI with EV-based microfluidic isolation, surface-enhanced Raman spectroscopy (SERS), fluorescence imaging, and multiomics analysis. Among these areas, AI-assisted EV diagnostic applications are comparatively closer to clinical translation, with several studies incorporating patient-derived samples and AI-assisted diagnostic platforms, whereas AI-guided therapeutic EV design strategies remain largely exploratory. With the continued accumulation of multicenter, cross-platform EV datasets, improvements in algorithmic robustness, and closer integration of computational and experimental workflows, AI may support further clinical evaluation of EV-based diagnostics and the systematic optimization of therapeutic EV platforms.

## 1. Introduction

Cancer remains one of the leading causes of mortality worldwide (Bray et al. 2024). Conventional tissue-based biopsies, while clinically informative, are invasive and insufficient for early screening or dynamic disease monitoring. As a result, clinical diagnostics are increasingly shifting toward liquid biopsy strategies, which are minimally invasive and more practical for longitudinal assessment (Tang et al. 2024). EVs are heterogeneous, lipid bilayer-enclosed particles spanning nano- to microscale dimensions and released by virtually all cell types. Acting as dynamic carriers of intercellular communication, EVs convey a molecular repertoire of proteins, lipids, and nucleic acids that mirror the functional and pathological status of their parental cells. EVs exhibit relative stability in body fluids and have emerged as promising biomarkers for noninvasive liquid biopsy applications (Kumar et al. 2024). EVs released by tumour cells actively contribute to cancer progression by enhancing cell proliferation, metastasis, angiogenesis, therapeutic resistance, and immune evasion, while their molecular signatures reflect tumour status and treatment response (Ye et al. 2024). These features confer unique advantages on EVs for cancer diagnosis, therapeutic monitoring, and recurrence surveillance (Yang et al. 2020).

Although notable progress has been made with biosensing approaches such as plasmonic biosensing platforms (Zhou et al. 2025), surface-enhanced Raman spectroscopy (SERS) (Ho et al. 2024), and microfluidics (Cong et al. 2024), their clinical translation remains hindered by the considerable heterogeneity of EV subtypes and the complexity of patient-derived samples. EV composition varies according to cellular origin, vesicle subtype, and physiological or pathological state, while differences in sample collection, processing, storage, and isolation procedures introduce additional pre-analytical variability (Chen et al. 2026; Suresh and Zhang 2026). These biological differences and technical sources of variability complicate the reliable detection and accurate interpretation of EV-associated molecular signals in complex biofluids. Current approaches are often hindered by difficulties in integrating multi-marker datasets, high background noise in patient-derived samples, and a lack of cross-platform standardisation (Mizenko et al. 2024). These challenges have prompted growing interest in AI-based analytical methods capable of extracting informative features from complex, high-dimensional EV data. AI-integrated microfluidic platforms may support EV capture, signal analysis, and classification, whereas AI-guided microbubble systems have primarily been explored for imaging and targeted delivery applications (Akbar et al. 2021; Chen et al. 2025). Deep learning approaches may facilitate the integration of realtime imaging, Raman spectroscopy, and multiomics data to support EV recognition and classification (Shin et al. 2023; Zhang et al. 2024; Al-Daffaie et al. 2025). Models based on machine learning for classification and temporal prediction show potential for multicancer early detection, dynamic therapeutic monitoring, and recurrence risk stratification (Diao et al. 2023; Shin et al. 2023; Hyon et al. 2025). Furthermore, data-driven design strategies may help predict the biodistribution, uptake pathways, and delivery efficiency of EVs, thereby informing the development of engineered delivery vehicles (Greenberg et al. 2023). Notably, intrinsic EV heterogeneity and pre-analytical variability remain important challenges affecting data consistency and result interpretation. At present, AI is therefore better positioned as a complementary analytical tool for feature extraction, integration, and interpretation of complex EV data. The reliability and application potential of AI-enabled EV research remain closely linked to the quality, clinical and biological diversity, and standardisation of the underlying datasets. Overall, AI may support EV research through multimodal data integration, diagnostic classification, temporal prediction, and the modelling of delivery-related behaviours.

## 2. Overview of tumour extracellular vesicles

**Figure 1.**
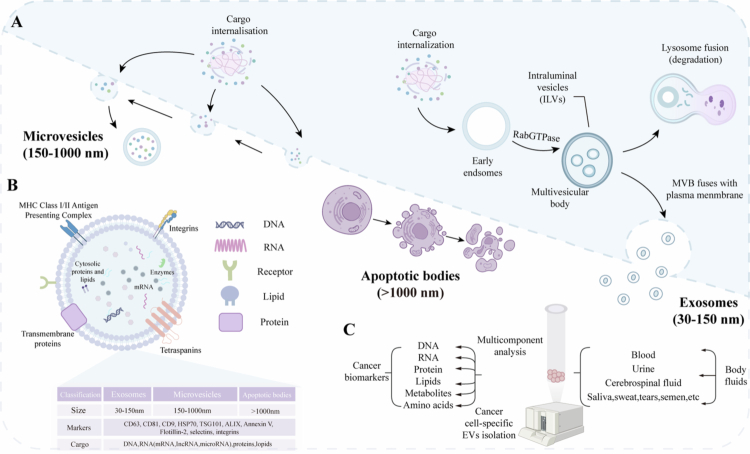
Overview of EVs. (A) Biogenesis and classification of EVs, including microvesicles, apoptotic bodies, and exosomes, based on their distinct formation pathways from endosomal compartments or the plasma membrane. (B) Size distribution and structural features of EVs, illustrating their nanoscale dimensions ranging from approximately 30 nm to 5000 nm and their lipid bilayer-enclosed morphology. (C) Molecular composition and functional roles of EVs, highlighting their cargo of proteins, lipids, and nucleic acids and their involvement in intercellular communication, disease progression, and therapeutic applications.

EVs are membrane-enclosed particles surrounded by a lipid bilayer and are widely distributed in diverse biological fluids, including plasma, urine, milk, and cerebrospinal fluid (CSF) (Kumar et al. 2024). They serve as mediators of intercellular communication by facilitating the transfer of bioactive molecules and signalling cues between cells (Tao and Guo 2020). Within the tumour microenvironment, EVs facilitate communication among tumour cells, stromal cells, and immune cells, playing important roles in tumour initiation, progression, and systemic regulation. Derived from parental cells, EVs partially retain the molecular and functional characteristics of their cells of origin and typically exhibit low immunogenicity and toxicity (Xia et al. 2024), which endow them with potential for applications in disease diagnosis, biomarker discovery, and therapeutic delivery. EVs constitute a heterogeneous population of vesicles spanning sizes from the nanoscale to the microscale (Hallal et al. 2022). Traditionally, EVs have been categorised based on size and biogenesis into three main subtypes: exosomes (30–150 nm), microvesicles (150–1000 nm), and apoptotic bodies (1000–5000 nm) (Wang et al. 2022). Exosomes are released through the fusion of multivesicular bodies with the plasma membrane (Heijnen et al. 1999), microvesicles are generated by direct outward budding of the plasma membrane (Ratajczak and Ratajczak 2020), and apoptotic bodies arise during programmed cell death through membrane blebbing and cellular fragmentation (Figure 1A). However, substantial overlap exists among these subtypes in terms of size, morphology, and molecular composition, and their biogenetic origins are often difficult to distinguish under routine isolation and characterisation conditions. To improve rigour and terminological consistency, a list of Minimal Information for Studies of Extracellular Vesicles (MISEV) recommends a size-based operational classification, defining small EVs (sEVs, < 100 nm or < 200 nm), which include exosomes and small microvesicles, and medium/large EVs (m/lEVs, > 200 nm), encompassing larger microvesicles and apoptotic bodies (Théry et al. 2018; Welsh et al. 2024). Given that most experimental preparations consist of mixed vesicle populations, this review adopts “EVs” as an umbrella term throughout.

EVs transport a broad spectrum of bioactive cargo, including proteins, lipids, DNA, RNA, and metabolites, thereby contributing to intercellular communication and regulation of the cellular microenvironment (Sheng et al. 2025) (Figure 1B). Proteomic studies have demonstrated that several membrane and cytosolic proteins, such as CD9, CD63, CD81, and TSG101, are enriched in EVs from diverse cellular origins. These molecules are therefore widely used as representative markers for EVs characterisation and enrichment (O’Brien et al. 2020). In tumour biology, EVs released from cancer cells serve as important mediators of molecular exchange between tumour cells and the surrounding stroma. By transferring oncogenic molecules and immune regulatory signals to recipient cells, they participate in reshaping the tumour microenvironment and support multiple aspects of cancer progression, including increased proliferation, migration, invasion, resistance to therapy, and distant metastasis (Ma et al. 2021; Fridman et al. 2022; Ten et al. 2024). At the mechanistic level, EVs can activate signalling pathways that favour cell growth, suppress anti-tumour immunity through the delivery of immune checkpoint molecules such as PD-L1, which inhibit T cell function (Ricklefs et al. 2018), and disseminate inflammatory or metastasis-related factors that contribute to extracellular matrix remodelling and immune regulation (Becker et al. 2016). Because of these properties, EVs are increasingly investigated as potential biomarkers and analytical targets for liquid biopsy and early cancer detection.

As naturally occurring membrane-bound particles in biological fluids, EVs can be detected in a variety of biofluids (Figure 1C), providing a foundation for tumour liquid biopsy and the development of non-invasive biomarkers. However, the physicochemical properties, molecular cargo, and functional activities of EVs are sensitive to pre-analytical variables (Alexandre et al. 2024). Haemolysis can release red blood cell-derived nucleic acids and membrane fragments, interfering with particle counting and molecular analyses (Marques-Garcia 2020). Inappropriate storage buffers or temperatures may lead to vesicle adsorption loss, membrane disruption, or RNA degradation. Repeated freeze-thaw cycles and delays in sample processing can induce vesicle fusion, aggregation, or the release of additional vesicles, thereby altering both EVs abundance and molecular composition (Gelibter et al. 2022). These factors can compromise the accuracy and reproducibility of proteomic, transcriptomic, and functional analyses. Therefore, standardised sample collection procedures, timely isolation and processing, and storage at low temperatures (e.g. −80 °C) while avoiding repeated freeze-thaw cycles are essential to preserve the structural integrity and molecular stability of EVs (Ahmadian et al. 2024). Notably, the lipid bilayer unique to EVs provides intrinsic protection for their cargo (Gill et al. 2019), conferring greater environmental resilience and circulatory stability compared with free proteins or nucleic acids, thereby supporting their feasibility and reliability for biomarker development and therapeutic delivery applications.

Owing to their intrinsic lipid bilayer structure and relative stability in biological fluids, EVs are considered promising drug delivery vehicles (Marcianti et al. 2025; Lu et al. 2024). They can provide stable encapsulation and transmembrane transport for anticancer agents, enhancing drug accumulation at tumour sites. However, natural vesicles still face limitations in cargo loading capacity, delivery efficiency, and targeting precision. In recent years, engineered vesicles have been developed through rational design and functional modification to further expand their delivery potential. Therapeutic agents can be introduced endogenously via pre-transfection of parental cells or genetic engineering approaches (Johnston et al. 2025), or exogenously after EVs isolation through incubation, ultrasound treatment, electroporation, or extrusion (Han et al. 2021; Roerig and Schulz‐Siegmund 2023; Mediratta et al. 2025). It should be noted that electroporation and ultrasound may introduce methodological artifacts, generating false positive signals (Singh et al. 2025), and can also disturb membrane structure, alter particle size distribution, or induce cargo aggregation, thereby affecting surface charge properties and increasing the risk of cargo leakage and residual reagent contamination. Therefore, the use of proper experimental controls is recommended, such as processing control samples containing only buffer or only cargo under identical physical conditions. In addition, strategies for targeted delivery and functional modification toward tumour tissues have attracted increasing attention. By introducing specific peptides, antibodies, polysaccharides, or nucleic acid aptamers onto the EV membrane, recognition of tumour-associated receptors can be enhanced, improving tissue selectivity and cellular uptake (Zhu et al. 2014). For example, iRGD modification can facilitate interaction with integrin avβ3, thereby increasing EVs accumulation at tumour sites and facilitate delivery to tumor tissues. (Liu et al. 2022) Collectively, these approaches improve EVs delivery efficiency and functional adaptability, further expanding their potential value in tumour diagnosis and therapy.

## 3. Artificial intelligence in medicine

In recent years, AI technologies have been increasingly applied in medicine, offering distinct advantages in analysing high-dimensional, heterogeneous, and dynamically evolving multimodal data (Zhang et al. 2022; Prelaj et al. 2024). AI is increasingly applied in medical imaging analysis, electronic health record (EHR) mining, multiomics integration, and drug development, facilitating disease diagnosis, prognostic evaluation, and therapeutic decision making through the identification of latent patterns (Luo et al. 2023; Reddy 2024; Tiwari et al. 2025). For example, convolutional neural network (CNN) based imaging algorithms can automatically segment lesions on CT or MRI scans, demonstrating diagnostic performance comparable to that of expert clinicians in the screening of gastrointestinal cancers (Zhou et al. 2025). Natural language processing (NLP) techniques can convert unstructured clinical notes into structured variables, enabling the extraction of information on medication patterns, complication risks, and disease progression to support data-driven clinical research and decision support frameworks (Ahn et al. 2021; An et al. 2025). Furthermore, AI has facilitated the integration of multiomics data, including genomics, transcriptomics, proteomics, and metabolomics, alongside drug repurposing, thereby improving the efficiency and reliability of biomarker discovery through the consolidation of heterogeneous datasets (Xu et al. 2022; Mohammadzadeh-Vardin et al. 2024).

**Figure 2.**
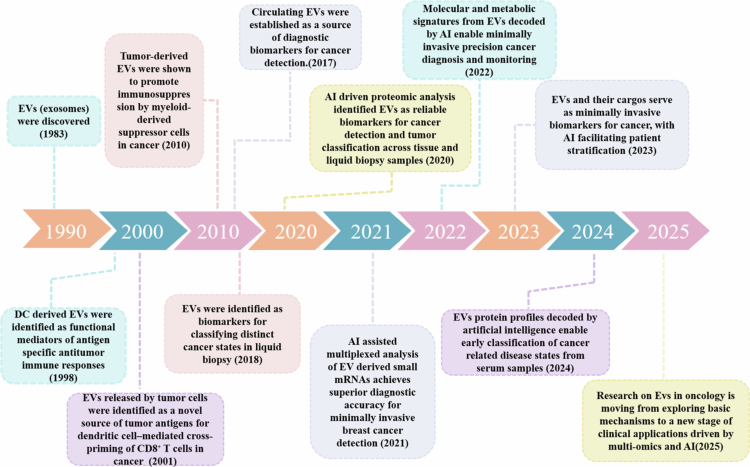
General overview of AI-EVs research in oncology. Timeline of key discoveries and milestone events in EVs research, biomarker development, and integration with AI for cancer detection, classification, and clinical translation.

As medical research has progressively advanced toward cellular substructures and molecular mechanisms, EVs have emerged as an increasingly important focus of investigation, attracting growing attention from the field of AI. Early studies on EVs primarily concentrated on their biological origins and functional characterisation. In 1983, vesicular trafficking associated with transferrin receptor recycling in reticulocytes was described, representing an early observation in the history of exosome discovery (Harding et al. 1983). Subsequently, in 1998, it was reported that EVs derived from dendritic cells could function as a cell-free antigen-presenting system capable of inducing antigen-specific antitumor immune responses, providing early evidence for the involvement of EVs in tumour immune regulation (Zitvogel et al. 1998). In 2001, further studies demonstrated that tumour-derived EVs could serve as a source of tumour antigens, be internalised by dendritic cells, and mediate cross-priming of CD8⁺ T cells, thereby reinforcing the functional link between EVs and tumour immune surveillance (Wolfers et al. 2001). By 2010, accumulating evidence indicated that tumour-derived EVs could promote immunosuppressive states by modulating the function of myeloid-derived suppressor cells, highlighting the immunoregulatory role of EVs within the tumour microenvironment (Chalmin et al. 2010). In 2017, machine learning approaches were combined with nanofluidic technologies to enable the diagnosis of pancreatic cancer using circulating EVs, marking an early convergence between EV-based biomarker research and AI methodologies (Ko et al. 2017). With the continued development of AI in medicine, AI technologies have been progressively incorporated into multiple critical stages of EVs research. After 2020, AI methods became more deeply integrated into EV studies. In 2021, AI-assisted quantitative analysis of EV-associated RNA significantly improved diagnostic accuracy in breast cancer, demonstrating the practical value of AI for the clinical translation of EV-based diagnostics (Liu et al. 2021). In 2022, AI-driven pattern recognition approaches were further applied to EV metabolic features, enabling effective discrimination between tumour and non-tumour samples (Chen et al. 2022). During 2023–2024, multiple studies employed AI-based systematic analyses of EV proteomic or spectroscopic data to achieve cancer type classification and early disease state identification (Parlatan et al. 2023; Yin et al. 2024). By 2025, with the deep integration of multi-omics technologies and AI, EVs research has progressively transitioned from a mechanism-oriented paradigm toward a clinically driven stage focused on translational applications (Greening et al. 2025) (Figure 2).

In the context of increasingly in-depth investigations into cellular substructures and molecular mechanisms, EVs, owing to their complex molecular signatures and diagnostic potential, have become an important focus of AI-driven biomedical research. AI technologies are increasingly applied to various dimensions of EVs research. In EV characterisation and classification, machine learning models integrate multimodal phenotypic data, including particle size distribution, zeta potential, and proteomic and lipidomic profiles, to accurately discriminate EV subtypes, trace their tissue origin, and predict disease-related alterations (Barlin et al. 2023; Xu et al. 2025). In cargo analysis, AI-based feature selection and pattern recognition algorithms are applied to identify diagnostic and prognostic biomarkers from EV miRNA, lncRNA, and circulating DNA, with multiple tumour-related studies reporting enhanced classification accuracy over conventional univariate approaches (Liu et al. 2025). In optimising delivery systems, AI may enable the simulation of critical parameters such as drug loading efficiency, membrane fusion probability, and targeting specificity, thus enabling the rational design of efficient and controllable EV-based therapeutic platforms. For high-throughput detection, combining deep learning with microscopy, Raman spectroscopy, or nanoscale flow cytometry offers great potential for rapid and label-free characterisation at the single vesicle level Liu et al. (2024).

## 4. AI-enabled strategies for extracellular vesicle-based cancer diagnosis and prediction

### 4.1 AI-driven extracellular vesicle analysis for early cancer detection and multicancer classification

**Figure 3.**
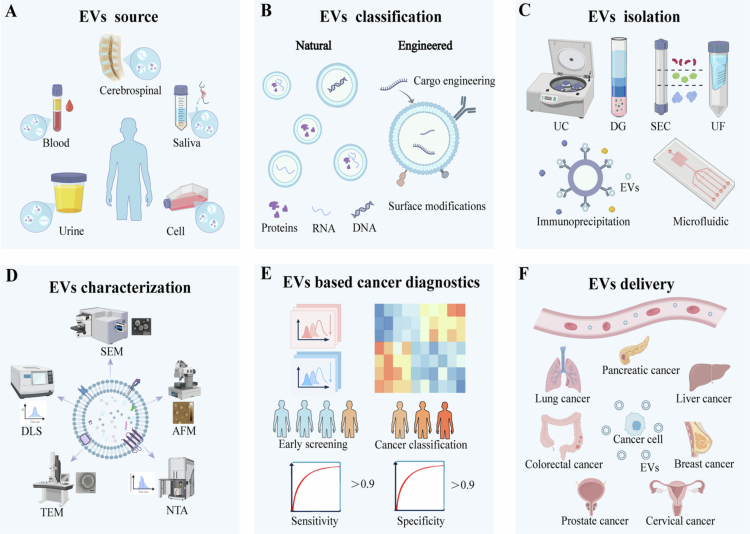
Tumour cell-derived EVs and their functional applications. (A) Common biospecimens used for EVs isolation include blood, cerebrospinal fluid, saliva, urine, and conditioned cell culture media. (B) Natural and engineered EVs, illustrating strategies for cargo loading and surface modification. (C) Representative EVs isolation workflows, comprising ultracentrifugation (UC), density gradient (DG), size-exclusion chromatography (SEC), ultrafiltration (UF), immunoprecipitation, and microfluidic platforms. (D) Core EVs characterisation techniques, including scanning electron microscopy (SEM), transmission electron microscopy (TEM), AFM, dynamic light scattering (DLS), and nanoparticle tracking analysis (NTA). (E) EV-based platforms for early cancer detection and diagnostic applications. (F) EVs derived from diverse tumour cell types, highlighting their potential as in vivo delivery vehicles.

AI has the potential to progress from serving as an auxiliary analytical tool to playing a more prominent role in data interpretation and hypothesis generation. Building on advances in medical imaging analysis, electronic health record processing, and multiomics integration, AI can identify informative patterns within high-dimensional, heterogeneous, and dynamically changing multimodal data, thereby supporting disease classification and risk prediction (Lin et al. 2024; Reddy 2024; Yang et al. 2025) (Table 1). In early cancer screening, the integration of multicomponent EV data with AI models facilitates the development of multicancer detection platforms (Shin et al. 2023). As relatively stable carriers in body fluids, EVs transport diverse molecular cargo, provide comprehensive multiparametric diagnostic information, and support longitudinal monitoring of tumour progression (Figure 3A, B). AI-driven multimodal data integration can facilitate the characterisation of EV heterogeneity and the extraction of disease-associated signals from complex biofluid backgrounds; however, it cannot fully eliminate the intrinsic biological variability of EV populations or the technical variation introduced during sample collection and processing.

**Table 1.** AI-assisted EVs for diagnostics.

Tumour type	Sample source	Sample size	Vesicle type	Method type	Accuracy	Major findings	
Lung, breast, colon, liver, pancreas, stomach cancer	Plasma	753 test samples (233 training and 520 test samples)	Exosomes (size-exclusion chromatography)	SERS + CNN-based AI classification	91.50%	Developed a label-free SERS-AI system enabling early-stage, multi-cancer detection and tissue-of-origin prediction with high accuracy; offers fast, cost-efficient, and scalable diagnostics.	Shin H. et al. (2023)
Prostate cancer (PIE-RADS 3 lesions)	Urine	102 blinded samples	Urinary exosomes (TMEM256, flotillin-2, PSMA)	Dual-gate FET + XAI	88.90%	XAI-guided multi-marker sensor doubles the diagnostic accuracy for PIE-RADS 3 lesions (66.7 % vs 30.4 %); non-invasive, interpretable PCa screening.	Choi J.Y. et al. (2025)
HER2⁺ breast cancer	Mouse urine	28 mice and 5 cell lines (HCC1954、HCC1143、MCF-7 MDA-MB-231 and the human fibroblast cell line)	Tumour urinary exosomes (HER2, GRB7)	SERS-tags + Deep Neural Network	/	Non-invasive, label-based SERS-DNN platform tracks trastuzumab efficacy by quantifying HER2/GRB7 on urinary exosomes; results correlate with qRT-PCR and tumour shrinkage.	Kim J. et al. (2024)
Paediatric medulloblastoma	CSF via extraventricular drainage (EVD)	12 CSF samples	Total CSF, microvesicles, exosomes, CPLL-enriched	WGCNA + SVM/PLS-DA + LC-MS/MS	96%	LMNB1 ↑ in MB vs controls (*p* < 0.001); SLC27A4 ↓ in MB. 192-protein core panel discriminates MB from non-tumour CSF, validated by ELISA.	Bruschi M. et al. (2022)
Colorectal, melanoma, prostate	Cell-culture supernatant	3 cell lines (COLO205、A375、LNCaP)	Cancer cell-derived exosomes (COLO205, A375, LNCaP)	Raman + PCA-LDA	93.3 % overall (F1 98.2 %/91.1 %/91.0 %)	Label-free Raman fingerprinting distinguishes exosome origin via PCA-LDA; lipid similarity analysis reveals cancer-type-specific lipidomes for non-invasive diagnostics.	Villazon J. et al. (2025)
Breast & cervical cancer	Human serum	17 clinical samples and 3 cell lines (H8, HeLa, and MCF-7)	Exosomes (label-free)	SERS + 3D AuNP nanomembranes + PCA-LDA	93.30%	Label-free SERS fingerprints enable fuzzy tumour vs. normal exosome discrimination; a model trained on H8/HeLa/MCF-7 spectra achieves 91.1% accuracy, 93.3% in clinical serum, and tracks DOX-induced molecular changes in MCF-7 exosomes over 0–32 h.	Diao X. et al. (2023)
Breast cancer (TNBC, Luminal A/B, HER2⁺)	Human serum	34 clinical samples and 4 cell lines (MDA-MB-231, MCF-7, BT474, and SKBR-3)	Exosomes (label-free)	SERS + Au-nanostar substrate + ANN deep learning	100 % (non-surgical)	ANN trained on four breast cancer cell exosomes achieves 100% subtype ID in non-surgical patients; PCA-based similarity (Dhealthy/Dcancer) assesses post-op outcomes, dropping to 0–0.2 after complete resection.	Xie Y. et al. (2022)
Colon & bladder cancer	Human plasma (1 drop, 40 µL)	110 clinical samples and 4 cell lines (A549, MCF-7, HeLa, and HepG2)	Circulating exosomes (PD-L1⁺/EpCAM⁺)	Aptamer-QN cascade assembly + XGBoost	83.8 % (7-feature model)	Dual-aptamer QN probes enable bicolour PD-L1/EpCAM phenotyping; 3-round DNA-scaffold amplification achieves LODs ≤ 31 ng/mL. XGBoost trained on 110 plasma samples (37 healthy, 38 colon, 35 bladder) reaches 83.8% accuracy and predicts immunotherapy response via exosomal PD-L1.	Zhang Y. P. et al. (2024)
Lung cancer (LUAD, LUSC, SCLC, AIS/MIA)	Human serum	643 participants	Circulating exosomes (label-free)	SERS (KI-AgNP film) + AutoGluon-ML + SHAP	100 % (Stage I LUAD)/81 % (AIS-MIA vs NLC)	643-participant cohort; iodide-modified AgNP substrates deliver high-SNR spectra from 0.5 µL serum exosomes; D2SE models achieve 100 % accuracy for Stage I LUAD, 81 % for precancerous lesions (AIS/MIA), 86 % balanced accuracy for LUSC, and 100 % for NSCLC vs SCLC subtyping; SHAP reveals lipid/protein spectral drivers.	Liu Y. et al. (2024)
Lung adenocarcinoma	Human plasma	84 clinical samples	Circulating exosomes	3D surround-enhancing SERS (3D se-SERS) + VGG-16 deep learning	86 % sensitivity/90 % specificity	Alginate-AuNP microsphere platform densely surrounds analytes with “hotspots,” enabling multiplex, label-free detection of 7 exosomal surface proteins (CD63/CD81/CD9/CD151/CD171/TSPAN8/PD-L1). VGG-16 model (7-class > 93 % accuracy) converts probability maps into colour barcodes, quantitatively distinguishing 20 healthy controls from 64 lung-cancer patients and tracking stage IA → IB → II progression (*p* < 0.001).	Chen M. et al. (2024)

Notes: “/’’ Representing no data; “↑” indicates increase, and “↓” indicates decrease.

Abbreviations: SERS, surface-enhanced Raman spectroscopy; CNN, convolutional neural network; AI, artificial intelligence; PI-RADS, prostate imaging reporting and data system; FET, field-effect transistor; XAI, explainable artificial intelligence; HER2, human epidermal growth factor receptor 2; DNN, deep neural network; CSF, cerebrospinal fluid; EVD, extraventricular drainage; WGCNA, weighted gene co-expression network analysis; SVM, support vector machine; PLS-DA, partial least squares discriminant analysis; LC-MS/MS, liquid chromatography–tandem mass spectrometry; qRT-PCR, quantitative reverse-transcription polymerase chain reaction; PD-L1, programmed death-ligand 1; EpCAM, epithelial cell adhesion molecule; ANN, artificial neural network; LOD, limit of detection; KI-AgNP, potassium iodide–modified silver nanoparticles; ML, machine-learning; SHAP, shapley additive explanations; SNR, signal-to-noise ratio; LUAD, lung adenocarcinoma; AIS, adenocarcinoma in situ; MIA, minimally invasive adenocarcinoma; LUSC, lung squamous cell carcinoma; NSCLC, non-small cell lung cancer; SCLC, small cell lung cancer.

SERS combined with nanointerface engineering can substantially amplify the molecular fingerprint signals of EVs, transforming EV-associated miRNAs and surface proteins into high-dimensional spectroscopic features suitable for machine learning-based classification (Lee et al. 2019; Chen et al. 2025) (Table 2). AI-based feature extraction and pattern recognition not only enable efficient and objective EV identification but also facilitate the automated classification of EV subpopulations and the analysis of their associations with disease phenotypes, thereby providing more reliable analytical tools for liquid biopsy applications. Rather than replacing conventional TEM, NTA, and DLS, AI-integrated approaches may complement these techniques by improving automated feature extraction, analytical sensitivity, and throughput (Lu et al. 2025).

**Table 2.** SERS-based analysis of EVs for cancer diagnosis.

Cancer type	EVs resource	Sample size	Purification	NP size (nm)	Data analysis	Successful rate	SERS substrate	SERS characteristics	Ref.
Multiple	Plasm	753 test samples (233 training and 520 test samples)	SEC	100	Deep learning	Cancer presence: AUC 0.970; sensitivity 89.4%; specificity 96.3%; Each TOO model AUC 0.925 on average	AuNP-aggregated array on APTES-functionalized glass	Being simple, nondestructive, requiring fewer analytes, and can be identified without labelling and specific antigens.	Shin H. et al. (2023)
Colorectal adenocarcinoma, melanoma, prostate	Cell	3 cell lines (COLO205, A375, and LNCaP)	Commercially purified	/	PCA-LDA	Overall accuracy 93.3%	/	Being noninvasive, nondestructive, label-free and requiring minimal sample preparation, high molecular specificity, and providing a detailed chemical fingerprint of the sample.	Villazon J. et al. (2025)
Breast, cervical	Serum, cell	17 clinical samples and 3 cell lines (H8, HeLa, and MCF-7)	Commercial assay kits	80	PCA-LDA	Cell-line exosomes total accuracy 91.1%, human serum exosomes total accuracy 93.3%	3D trilayer AuNP nanomembrane transferred onto ITO glass	Achieving real-time monitoring of dynamic biological processes.	Diao X. et al. (2023)
Breast	Serum, cell	34 clinical samples and 4 cell lines (MDA-MB-231, MCF-7, BT474, and SKBR-3)	SEC + ultrafiltration	71.0 ± 9.6	Deep learning	100% accuracy for non-surgery patients; overall accuracy 79.4% when postoperative cases are included	An Au nanostar monolayer onto a solid substrate	Label-free SERS enables molecular detection directly in complex biological matrices.	Xie Y. et al. (2022)
Lung	Plasma	84 clinical samples	Commercial assay kits	50	Convolutional Neural Network	Sensitivity 86%, specificity 90%; stage I 85%, stage IA 82%	Analytes were embedded into an alginate-based microsphere together with abundantly well-distributed AuNPs to form 3D hotspots	Richer peaks and higher intensity than 2D AuNP plate; plasma exosome spectra reveal Raman bands from proteins and lipids.	Chen M. et al. (2024)
Lung	Cell	4 cell lines (BEAS-2B, NCI-H460, NCI-H226, and PC-9)	Commercial assay kits	70	Convolutional Neural Network	Overall accuracy 97.88%	Microfluidic SERS chip with size-based trapping and PACD immuno-capture probes	High sensitivity, high reproducibility and high specificity.	Chen H. et al. (2025)
Breast	Serum	28 clinical samples	Immunomagnetic capture	/	PCA-LDA-SVM machine learning	Label-free Raman: AUCs > 89%; HER2-MEDN: accuracies > 94%	Magnetic beads@HER2-Exos@HER2-SERS detection nanoprobes	High diagnostic accuracy	Jia Y. et al. (2025)

Note: “/’’ Representing no data.

Abbreviations: AUC, area under the curve; TOO, tissue of origin; PCA-LDA, principal component analysis–linear discriminant analysis; ITO, indium tin oxide; SEC, size-exclusion chromatography; PACD, platform utilized multilayer gold nanocubes; SVM, support vector machine.

For example, Shin et al. developed an Exosome-SERS-AI liquid biopsy platform that applies deep learning to the SERS profiles of plasma exosomes for the simultaneous detection of six types of early-stage cancer (Shin et al. 2023). In 520 test samples that had not been used for model training, the system achieved an AUC of 0.970 for cancer presence detection. Among 278 patients with early-stage cancer, the mean AUC for tissue-of-origin (TOO) discrimination was 0.945. The final integrated decision model showed a sensitivity of 90.2% at a specificity of 94.4% and correctly predicted the tissue of origin in 72% of positive patients. However, the study used a retrospective design, and the test cohort comprised 360 cancer cases and 160 healthy controls. The six cancer types were also unevenly represented, with 100 lung, 70 breast, 70 colorectal, and 40 each of liver, pancreatic, and stomach cancer cases. The reported overall performance may therefore have been influenced by the composition of the cohort and may not fully reflect performance in the less represented cancer groups. Moreover, the use of selected healthy controls differs from population-level screening, which also involves individuals with benign tumours, inflammatory diseases, and other comorbidities that may affect exosome-associated signals.

Similar concerns arise from an EV protein-based blood biomarker classifier developed to detect stages I and II pancreatic, ovarian, and bladder cancer (Hinestrosa et al. 2022). In this case-control pilot study, 139 pathologically confirmed stage I and II cancer cases were compared with 184 control subjects, yielding an overall AUC of 0.95. However, sensitivity for stages I and II combined varied considerably by cancer type, ranging from 43.8% for bladder cancer to 75.0% for ovarian cancer and 95.7% for pancreatic cancer. Thus, a high overall AUC may obscure substantial differences in performance among individual cancer types. Choi et al. subsequently developed an XAI-based prostate cancer screening system integrating a dual-gate field-effect transistor-based multi-marker biosensor with the sensing patterns of three urinary exosomal biomarkers, TMEM256, flotillin-2, and PSMA (Choi et al. 2025). The system achieved an AUC of 0.93 in 102 blinded samples, and the diagnostic accuracy for PIE-RADS 3 lesions was more than twice that obtained using conventional PIE-RADS scoring alone. The XAI analysis further identified TMEM256 as the leading factor in screening patients with PIE-RADS 3 lesions. Nevertheless, the relatively small and selected clinical cohort limits the extent to which these findings can be generalised to broader screening populations.

More recently, EV-AI studies have begun to adopt more rigorous validation designs. Wang et al. developed and prospectively validated an EV protein-based blood test for the preclinical detection of oesophageal squamous cell carcinoma (ESCC) (Wang et al. 2026). The biomarker panel included EV-associated squamous cell carcinoma antigen (SCC) and matrix metalloproteinase-13 (MMP13), which were integrated with clinical factors into an interpretable multi-criteria decision-making classification fusion (MCF) machine-learning framework. The MCF model was trained and validated in prospective, multicentre diagnostic cohorts comprising 1,018 participants, and its preclinical detection capability was subsequently assessed in a prospective, population-based longitudinal cohort. Although this study focused on a single cancer type, its prospective, multicentre, external-validation, and longitudinal design provides stronger evidence for the clinical evaluation of EV-based machine learning models. Nevertheless, such validation remains uncommon, particularly for multicancer EV-AI platforms. Retrospective discrimination between known cancer cases and selected controls differs substantially from population-level screening, where cancer prevalence is lower and benign diseases and comorbidities are more common. Under these conditions, false-positive findings and positive predictive value become particularly important. Therefore, the high AUCs and accuracy values reported in current studies should be interpreted in the context of cohort size, class distribution, control-group composition, and validation design (Collins et al. 2024; Sounderajah et al. 2025). Population-level screening evidence for multicancer EV-AI platforms remains limited, and their clinical utility requires further evaluation in large, prospective, and population-representative cohorts.

### 4.2 AI in dynamic monitoring of extracellular vesicles

During treatment, EVs, nanoscale carriers of information reflecting disease progression and immune status, exhibit dynamic changes in their molecular profiles. Tumour-derived EV can facilitate the intercellular transfer of resistance-associated molecules, endowing recipient cells with drug-resistant phenotypes. Notably, temporal fluctuations in exosomal PD-L1 levels during therapy may serve as predictive biomarkers for sustained immune responses, offering a valuable signal for realtime monitoring of treatment efficacy (de Miguel-Perez et al. 2022). The primary role of AI is to perform high temporal resolution, multidimensional analysis of EV-associated signals to support realtime assessment of therapeutic efficacy and prediction of immune responses. For example, Kim et al. developed a deep learning assisted SERS immunoassay to monitor treatment responses in HER2 overexpressing breast cancer mouse models (Kim et al. 2024). In this study, four types of gold@silver (Au@Ag) nanoparticles carrying distinct Raman reporter molecules were prepared and functionalized with antibodies targeting EV-associated biomarkers (GRB7, CD63, GAPDH, or HER2). This platform captures composite molecular fingerprints, in which the resulting signals are influenced by the combined contributions of reporter-tagged immunoassay components and the underlying biochemical composition of EVs. By applying deep learning algorithms, the system successfully resolved and analysed complex spectral signatures derived from these reporter tags. When applied to urinary EV samples in mouse models, this approach enabled dynamic tracking of therapy-associated changes in SERS spectral features associated with EV surface antigen-related signals, particularly those linked to HER2 and GRB7. Following trastuzumab treatment, a significant attenuation in SERS spectral intensities associated with HER2- and GRB7-related features was observed, demonstrating the system’s capability to monitor therapeutic responses by interpreting composite molecular changes rather than direct biological quantification.

EVs are increasingly recognised for their value in liquid biopsy applications, extending beyond therapeutic monitoring to the assessment of disease recurrence risk. In medulloblastoma, a machine learning-based framework has been established to analyse EV-associated proteomics from CSF (Bruschi et al. 2022). Using liquid chromatography tandem mass spectrometry, a total of 3,560 proteins were identified in EVs from CSF samples of both medulloblastoma patients and controls. Weighted gene co-expression network analysis (WGCNA) clustered these proteins into nine modules. Notably, a protein module closely associated with medulloblastoma, comprising 1,276 proteins, exhibited a correlation coefficient of 0.8, whereas a module corresponding to control samples, containing 29 proteins, showed a correlation coefficient of 0.7. Integrating support vector machine (SVM) learning with partial least squares discriminant analysis (PLS-DA), 192 core protein biomarkers were ultimately selected, among which exosomal long-chain fatty acid transport protein 4 (SLC27A4) and lamin B1 (LMNB1) demonstrated significant discriminative capability. These findings suggest that the combined use of EV protein biomarkers and machine learning algorithms represents a promising emerging strategy for liquid biopsy, with potential utility in early diagnosis and recurrence risk prediction in medulloblastoma.

Beyond static sample analysis, dynamic assessment of therapeutic efficacy has emerged as a critical area for AI integration in EV-based diagnostics. In one study, a Raman spectroscopy-based dynamic monitoring model extracted spectral features of cancer-derived EV using principal component analysis (PCA) and combined them with linear discriminant analysis (LDA) to classify EV from colorectal cancer (CRC), melanoma, and prostate cancer cell lines, achieving an accuracy of 93.3% (Villazon et al. 2025). Building on this, time series analysis algorithms were applied to model temporal changes in EV-associated spectral features, enabling realtime monitoring of EV composition during tumour treatment. This dynamic approach enables the characterisation of temporal patterns in EV expression profiles and improves sensitivity to therapy-associated changes, thereby offering valuable insights for longitudinal monitoring and early translational research. Similarly, another study employed label-free SERS combined with machine learning, using a three-layer gold nanoparticle membrane enriched with “hot spots” as a substrate to classify EV samples derived from different cell lines based on composite SERS spectral fingerprints (Diao et al. 2023), including normal H8 cells and cancerous HeLa and MCF-7 cells, with a predictive accuracy of 91.1%. When applied to clinical serum samples from breast and cervical cancer patients, the model demonstrated an improved predictive accuracy of 93.3% in this exploratory cohort. Extending this approach to chemotherapy monitoring, doxorubicin (DOX) was used as a model chemotherapeutic agent to assess temporal changes in EV-derived composite SERS spectral features secreted by MCF-7 cells at 0, 8, 24, and 32 h post-treatment, providing a novel framework for realtime assessment of chemotherapy efficacy.

Beyond Raman and SERS-based approaches, Fourier-transform infra-red (FTIR) spectroscopy has recently emerged as a complementary, label-free modality for EV-based liquid biopsy. Wong et al. combined urinary EV FTIR spectra with clinical variables using a linear SVM framework. Following feature selection and SMOTE-based class balancing, the integrated model achieved an AUC of 0.92 for prostate cancer detection, outperforming conventional PSA-based screening (Wong et al. 2025). Similarly, Di Santo et al. employed an unsupervised autoencoder to compress blood-derived EV ATR-FTIR spectra into a low-dimensional latent space. Notably, a single latent feature encoding Amide II and lipid CH₂ vibrational modes discriminated hepatocellular carcinoma patients from cirrhotic controls with AUC of 0.785, a performance comparable to that of serum *α*-fetoprotein (Di Santo et al. 2025). FTIR captures global vibrational signatures arising from lipids, proteins, and nucleic acids within intact EV, providing biochemical information largely complementary to Raman scattering. Collectively, these proof-of-concept studies underscore the translational potential of FTIR-based EV spectroscopy when coupled with modern AI pipelines, and suggest that multimodal vibrational workflows integrating Raman and infra-red readouts may enhance the accuracy and robustness of next-generation liquid biopsy platforms.

### 4.3 AI-driven modelling of tumour heterogeneity and progression through extracellular vesicles

For stratified diagnosis of tumour heterogeneity, a study proposed a deep learning-based SERS approach. Using an artificial neural network (ANN) algorithm, the model was trained on SERS spectral features derived from cancer cell-derived EV samples, achieving accurate classification of different breast cancer subtypes with a predictive accuracy of 100% (Xie et al. 2022). By extracting discriminative features from the molecular information encoded in SERS spectra and associating them with breast cancer subtype-related molecular patterns, the model demonstrated the potential of EV-derived composite spectral signatures for breast cancer subtype classification. Similarity analysis combined with PCA further enabled the assessment of postoperative outcomes in breast cancer patients, suggesting that this approach may have potential value in EV-based precision diagnosis and treatment monitoring. However, these findings were obtained under the specific conditions of the original dataset and validation strategy. Given the high dimensionality of EV-derived spectral profiles and the relatively limited sample size commonly encountered in biomarker studies, such models may be susceptible to overfitting, whereby models may capture cohort-specific variations or noise rather than reproducible disease-related patterns (Kim et al. 2026). Therefore, the reported performance should be considered in the context of the original study design and validation process, and further evaluation in larger, independent, and clinically diverse cohorts is needed to determine the reproducibility and broader applicability of these findings (Collins et al. 2024).

AI-assisted EV detection and analysis have also contributed to advances in liquid biopsy and immunotherapy response prediction. Zhang et al. developed a signal-amplification strategy based on aptamer recognition and DNA scaffold hybridisation-triggered assembly of quantum dot nanospheres (QNs), enabling bicolour fluorescence phenotyping of EV-associated PD-L1 and EpCAM in conjunction with machine learning (Zhang et al. 2024). By employing a DNA scaffold-assisted QN assembly strategy, the method accurately distinguished colon cancer, bladder cancer, and healthy individuals. Through multiple rounds of signal amplification, the technique demonstrated high sensitivity and specificity in the quantitative analysis of EV-associated PD-L1, achieving a detection limit as low as 24 ng/mL. Dual signal detection revealed that EV-associated PD-L1 and EpCAM levels were significantly elevated in cancer patients compared to healthy controls, with ROC analysis confirming their validity as cancer biomarkers, with AUCs of 0.9533 and 0.9321, respectively. By integrating EV-associated PD-L1 and EpCAM fluorescence features using machine learning, the study successfully enabled early cancer screening and prediction of immunotherapy response. In a cohort of 78 participants comprising healthy donors and patients with cancer, the model achieved an overall accuracy of 83.84%. This strategy, combining QNs signal amplification with machine learning, not only simplified sample pre-processing but also significantly improved detection precision. By extracting key features from high-dimensional EV-associated expression matrices and linking them to immune phenotypes, this approach provides a robust foundation for immune response prediction based on EV-associated cargo. Notably, many AI-based approaches characterise tumour heterogeneity and disease progression by learning molecular information encoded in EVs (Hoshino et al. 2020; Tiwari et al. 2026). In such settings, the dimensionality of EV-derived molecular profiles often substantially exceeds the available sample size, which increases the risk of overfitting if model development and validation are not carefully designed (Ansermino et al. 2026). Previous studies in proteomics have demonstrated that conventional cross-validation can yield overly optimistic performance estimates when feature selection or hyperparameter tuning is conducted outside the validation loop, thereby limiting generalisability to independent clinical cohorts (Ivarsson Orrelid et al. 2025). To address this issue, recent studies increasingly adopt nested cross-validation to enable an unbiased estimation of predictive performance on unseen data, biomarker stability analysis under resampling to evaluate the reproducibility of selected EV-associated features, and validation in independent cohorts to assess model transferability across patient populations and analytical platforms (Tiwari et al. 2026). Collectively, these strategies support more reliable evaluation of AI models for EV-based characterisation of tumour heterogeneity and disease progression.

A machine learning-assisted SERS analysis strategy employing iodide-modified silver nanofilms has been developed to analyse composite SERS spectral fingerprints associated with serum EV samples from lung cancer patients (Liu et al. 2024). By integrating interpretable machine learning algorithms, this approach was reported to achieve 100% accuracy in detecting stage I lung adenocarcinoma within the analysed cohort and to distinguish different lung cancer subtypes and disease stages. These findings suggest that EV-derived spectral fingerprints may provide valuable molecular information for lung cancer classification and patient stratification. However, the reported performance should be further evaluated across independent cohorts, as differences in patient characteristics, sample composition, and analytical workflows may influence the model reproducibility and broader applicability (Moons et al. 2025). Further validation in prospectively collected clinical cohorts will help determine whether these findings can be consistently reproduced across different populations and analytical settings (Tiwari et al. 2026). Meanwhile, Chen et al. proposed a three-dimensional surround-enhanced SERS (3D se-SERS) multiplex protein analysis platform to profile composite spectral signatures associated with the expression patterns of seven EV surface proteins (Chen et al. 2024). Together, these studies highlight the potential of EV-based SERS approaches for molecular characterisation and noninvasive assessment of lung cancer. Collectively, AI-driven EV-based diagnostics and therapeutic strategies have established a coherent framework spanning early detection, risk prediction, disease staging, treatment response evaluation, and progression monitoring (Li et al. 2024). Throughout this process, AI methodologies have evolved from conventional machine learning approaches, such as random forest (RF) and SVM, to deep learning models, including CNN, long short-term memory networks (LSTM), and autoencoders, and further to emerging techniques such as attention mechanisms, multitask learning, and self-supervised learning. These models have demonstrated continuous advances in feature selection, temporal modelling, data integration, and heterogeneity stratification. Future efforts should focus on enhancing model interpretability, improving cross-platform data adaptability, and optimising clinical integration workflows to facilitate the translation of these technologies into EV-driven precision oncology.

## 5. AI enhanced extracellular vesicle isolation and characterisation

### 5.1 AI-based approaches for extracellular vesicle identification and classification

AI has demonstrated effective adaptability and computational power in target recognition, feature extraction, and classification tasks, particularly in handling multidimensional and heterogeneous datasets, thereby enhancing both the accuracy and efficiency of EV isolation (Lin et al. 2024) (Table 3). Beyond enabling efficient isolation, the identification and classification modelling of EVs has emerged as another critical application of AI technologies. In one of the early explorations in this field, Ito et al. employed atomic force microscopy (AFM) to capture morphological data of EVs derived from three cancer cell lines, including size, shape, and solid-surface deformation (Ito et al. 2018). By integrating PCA with SVM, they developed a classification model that achieved a prediction accuracy of 85.2% in binary classification tasks and 82.6% in ternary classification. Notably, PCA revealed that the relationship between particle volume and aspect ratio represented a key determinant influencing classification performance.

**Table 3.** AI-assisted EVs isolation.

Tumour type	Sample source	Vesicle type	Method type	Accuracy	Major findings	Ref.
Non-small cell lung cancer (NSCLC: LUAD, LUSC, LCC)	Cell-culture supernatant	Exosomes (30–150 nm)	Microfluidic-SERS + CNN-ResNet	97.88	Label-free, high-throughput platform enabling precise NSCLC subtyping via interpretable SERS spectral patterns.	Chen H. et al. (2025)
HT-29 (colorectal), HT-1080 (fibrosarcoma), MIA PaCa-2 (pancreatic)	Cell-culture supernatant	Exosomes (30–150 nm)	AFM + SVM (RBF kernel) + PCA	85.2 % (2-class), 82.6 % (3-class)	Morphological deformation on solid surfaces enables label-free host-cell prediction; TiO₂ substrate yields highest accuracy.	Ito K. et al. (2018)
Pan-cancer (breast, colorectal, glioblastoma, lung, liver, neuroblastoma, pancreatic, etc.)	Plasma/Serum/Urine/Tissue	Exosomes (~40–180 nm)	Random-Forest classifier on proteomics	90–95 %	18-plasma/serum, 5-cancer-type, 17-urinary protein panels enable universal, high-accuracy cancer exosome detection across fluids.	Li B. et al. (2024)
Breast & pancreatic cancer	Human plasma (220 clinical samples)	Plasma exosomes (~50–150 nm)	SSEC isolation → MALDI-TOF MS → Exo-ANN deep learning	80 %	Rapid (<2 h) high-purity exosome isolation + 10-feature ANN achieves 80 % accuracy, outperforming LR/DT/KNN/SVM, enabling non-invasive dual-cancer diagnosis.	Zheng H. et al. (2022)
Ovarian cancer	Cell-culture supernatants (SKOV3, HEY, A2780) & patient tissue	Exosomes (CD9⁺/CD63⁺⁺/CD81⁺/EPCAM⁺/HSP70⁺) & cytokines (IL-6/IL-8)	GOQD antibody-barcoded microfluidic chip + fluorescence immunoassay	99 %	First single-cell platform simultaneously profiling 5 exosome phenotypes + 2 cytokines; identifies EPCAM⁺/HSP70⁺ high-secretor tumour sub-clusters invisible in bulk analysis.	Song F.T. et al. (2022)
Pancreatic/breast cancer	Human serum (healthy, pancreatic, breast cancer)	Exosomes (30–100 nm, CD63⁺/CD81⁺/HER2/GPC-1/EpCAM/EGFR)	DNA-PAINT + qPAINT + LDA machine learning	100 %	Multiplex quantification of four surface biomarkers at single-exosome level enables 100 % accurate discrimination of pancreatic vs breast vs healthy exosomes; single-marker (GPC-1) fails.	Chen C. et al. (2019)
HER2-positive breast cancer	Patient serum (pre- & post-2-cycle neoadjuvant therapy)	HER2-positive exosomes (50–200 nm)	Raman/SERS spectroscopy + PCA-LDA-SVM	94%	Non-invasive Raman/SERS profiling of HER2-exosomes predicts NAT efficacy early; HER2-MEDN capture system boosts accuracy to > 94 % vs > 89 % with label-free Raman.	Jia Y.N. et al. (2025)
Breast cancer (MDA-MB-231)	Conditioned medium & EVs	Small RNAs (18–50 nt) in EVs	ExoGRU-GRU deep-learning model + exoCLIP + CRISPRi	95 % AUC	ExoGRU identifies YBX1, HNRNPA2B1, and RBM24 as regulators of small RNA secretion and enables the generation of synthetic small RNAs with high secretion ability.	Zirak B. et al. (2024)
Prostate cancer	First-catch first-morning urine	Urinary exosomes (50–200 nm)	EVLatch isolation + RT-qPCR (PCA3/HOXC6/DLX1 vs SPDEF) + Random-Forest AI (PURE-AID)	61.3 %	PURE-AID non-invasively distinguishes high-grade from low-grade PCa, cuts 54 % unnecessary biopsies; EEF1A1 serves as endogenous QC for urinary exosome quantification.	Qiu S. et al. (2025)

Abbreviations: LCC, large-cell carcinoma; ResNet, residual network; AFM, atomic force microscopy; RBF, radial basis function; PCA, principal component analysis; SSEC, sequential solid-phase extraction; MALDI-TOF MS, matrix-assisted laser desorption/ionisation time-of-flight mass spectrometry; LR, logistic regression; DT, decision tree; KNN, k-nearest neighbour; LDA, linear discriminant analysis; GOQDs, graphene oxide quantum dots; NAT, neoadjuvant therapy; GRU, gated recurrent unit; exoCLIP, exosomal cross-linking immunoprecipitation; CRISPRi, clustered regularly interspaced short palindromic repeats interference; EVLatch, extracellular vesicle latch; PCA3, prostate cancer antigen 3; HOXC6, homeobox C6; DLX1, distal-less homeobox 1; SPDEF, SAM pointed domain-containing ETS transcription factor; QC, quality control.

Beyond morphological recognition, classification modelling based on proteomic features of EV has also achieved significant breakthroughs. By leveraging EV proteomic signatures, researchers developed an RF-based classification system capable of distinguishing between cancer types and biological fluids. Proteins such as CLTC, EZR, and TLN1 were identified as key features and integrated into a pan-cancer biomarker panel (Li and Kugeratski, 2024). The resulting model demonstrated outstanding diagnostic performance, with AUC of up to 0.96 across multiple sample types and as high as 0.99 in independent validation cohorts, clearly outperforming conventional classifiers such as k-nearest neighbour (k-NN) and Naïve Bayes (NB). These findings underscore the advantage of AI in integrating multisource data and enhancing diagnostic accuracy, thereby supporting more systematic EV-based analytical interpretation, identification, and detection strategies with enhanced translational relevance. At the same time, substantial progress has been made in the automation of EVs detection. Deep learning has been applied to optimise microscopic image analysis, significantly improving the classification accuracy of scanning probe microscopy (SPM) images (Park et al. 2025). This advancement not only increased the efficiency and reliability of EV image-based analysis and interpretation after isolation but also reduced manual errors and analytical time, thereby supporting the development of more scalable and clinically relevant analytical workflows. For instance, Jin et al. introduced a deep learning-enhanced SPM imaging acceleration strategy for nanoparticle recognition. By integrating single particle interference scattering models with deep learning, they generated hybrid datasets and constructed a neural network based on the EfficientNet architecture (Jin et al. 2024). Their results demonstrated that this approach effectively classified interference scattering images and accurately identified multiple particles even under noisy conditions. By bridging the gap between simulated and experimental data and overcoming the limitations of small experimental datasets, this automated SPM image analysis pipeline achieved high-precision particle recognition under real-world conditions, paving the way for efficient, automated nanoparticle and EVs analysis in biomedical research.

### 5.2 AI-based optimisation and platform development for extracellular vesicle isolation

EVs have attracted growing attention in early cancer diagnosis owing to their rich molecular cargo and excellent stability (Lee et al. 2023). However, the first challenge in employing EVs for cancer research lies in their isolation and characterisation, as current separation processes face multiple technical bottlenecks (Jia et al. 2022) (Figure 3C, D). Conventional approaches, such as ultracentrifugation, density gradient centrifugation and size exclusion chromatography, often exhibit low efficiency and limited purity when processing complex biofluids (Visan et al. 2022; Zhang et al. 2022; Yan et al. 2023), Co-precipitation typically yields low purity and requires prolonged processing with relatively large sample volumes (Liu et al. 2025), ultrafiltration is prone to soluble protein contamination (Tavukcuoglu et al. 2024), and immunoprecipitation often has low yield and can bias downstream analyses (Rasuleva et al. 2023). The intrinsic heterogeneity of EVs, arising from diverse cellular origins and reflected in variations in size, charge, and density, poses a major obstacle to the reproducibility and reliability of downstream molecular and functional analyses (Carney et al. 2025). To overcome these limitations, researchers have been developing innovative separation platforms that enable higher sensitivity, faster processing, and reduced sample requirements. Microfluidic chip technologies, integrating nanopore arrays and surface functionalization strategies, allow for efficient capture and realtime analysis of nanoscale EVs from small volume samples (Li et al. 2023). Acoustic-based nanosorting approaches, leveraging ultrasonic standing waves, achieve size-selective separation while preserving vesicular integrity (Rufo et al. 2024). Despite these advances, most emerging isolation techniques still lack the adaptive capability to handle heterogeneous biological samples and largely depend on manual parameter optimisation (Table 4).

**Table 4.** Techniques for EVs isolation.

Technique	Purity	Equipment	Time	Yield	Specimen volume	Vesicle quality	Ref.
Ultracentrifugation (UC)	Low	Ultracentrifuge	≥210 min	Low	High (150 mL)	Vesicles damage	Visan K.S. et al. (2022)
Tangential Flow Filtration (TFF)	Low	Minimate Tangential Flow Filtration Capsules + peristaltic pump	≥130 min	High	High (150 mL)	Contained albumin	
Density Gradient Centrifugation (DGC)	Median	Ultracentrifuge + sucrose/iodixanol	>12 h	Low	Median (2 mL)	Vesicle damage	Zhang J et al. (2022)
Size-Exclusion Chromatography (SEC)	High	SEC column	≤8.5min	Low	Median (0.5–1 mL)	Preserves vesicle morphology	Yan S et al. (2023)
Sequential size-exclusion chromatography (SSEC)	High	Mini-SEC column + High-performance liquid SEC system	<2 h	Median	Low (500 μL)	Preserves vesicle morphology	Zheng H et al. (2022)
Co-precipitation (PEG)	Low	Polymer precipitation reagent	>14 h	Low	High (1 mL)	Preserves vesicle morphology	Liu W et al. (2025)
Ultrafiltration (UF)	Median	Ultrafiltration Membrane	<15 min	High	Low (100 μL)	Soluble protein contamination	Tavukcuoglu Z. et al. (2024)
Immunoprecipitation	High	Magnetic materials	<1.5 h	Low	Low (200 μL)	Bias the downstream analysis	Rasuleva K et al. (2023)
Microfluidics	High	Microfluidic chip	10 min	Low	Low (20 μL)	Preserves vesicle morphology	Li P et al. (2023)
Flocculation via orbital acoustic trapping (FLOAT)	High	Focused IDTs + function generator + amplifier	<10 min	High	Low (200 μL)	Less selective when separating vesicles from lipoproteins	Rufo J et al. (2024)

Abbreviations: UC, ultracentrifugation; TFF, tangential flow filtration; DGC, density gradient centrifugation; PEG, polyethylene glycol; UF, ultrafiltration; IDTs, interdigitated transducers; FLOAT, flocculation via orbital acoustic trapping.

**Figure 4.**
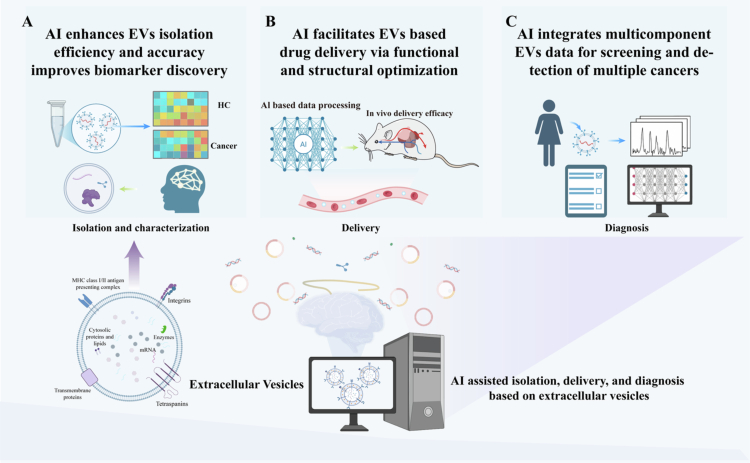
AI-assisted EVs isolation, delivery, and diagnostic applications. (A) AI-enabled optimisation of EVs isolation and characterization workflows, illustrating the integration of machine learning algorithms to improve separation efficiency, feature extraction, and data quality across diverse analytical platforms. (B) AI-driven design and optimisation of EV-based drug delivery systems, highlighting the use of computational models to guide vesicle functional profiling, surface engineering, and in vivo delivery performance. (C) AI-powered EVs diagnostic frameworks for cancer detection, illustrating the integration of multidimensional EV-derived molecular features for early cancer screening, disease classification, and multi-cancer detection.

To address the inherent limitations of conventional EV isolation methods, researchers are increasingly developing optimised physical isolation platforms that improve separation efficiency and sample handling, while enabling more reliable downstream analytical characterisation. For instance, a diffusion-based EV isolation chip employing a polycarbonate nanoporous membrane with 200 nm pores positioned between two chambers has enabled selective filtration of EVs from cell culture media and human serum (Park et al. 2025). Compared with traditional ultracentrifugation, this approach achieved a higher EV recovery rate while preserving vesicle integrity and functionality without requiring complex equipment or extensive preprocessing. As the demand for enhanced separation performance grows, integrated microfluidic platforms, together with AI-assisted downstream analysis, are emerging as a key direction for EV research. When combined with AI, these platforms are evolving into powerful diagnostic tools with substantial translational potential (Aubry et al. 2023) (Figure 3E). Li et al. developed a 3D porous sponge-based microfluidic chip platform that increased EV capture efficiency to nearly 90%. By integrating deep mass spectrometry and multistage screening, they identified SORL1 as a colorectal cancer (CRC) specific EV membrane protein and established a quantum dot-based detection strategy for SORL1 (Li et al. 2023). Building on this, they designed an AI-driven classification system that extracted features from 64 fluorescence images, achieving patient identification with an area under the ROC curve (AUC) of 0.99, markedly outperforming the conventional CEA biomarker (AUC = 0.71). Recently, Chen et al. incorporated deep learning into a microfluidic platform integrated with surface-enhanced Raman scattering (SERS) technology for the analysis of composite SERS spectral signatures associated with EV-derived from non-small cell lung cancer (NSCLC) samples. This platform used a composite capture unit comprising polystyrene microspheres conjugated with gold nanocubes and anti-CD9 antibodies (denoted as PACD) to enrich EVs, achieving a maximum trapping efficiency of approximately 85%. By interpreting these complex molecular fingerprints, which reflect the combined contributions of multiple biomolecular components, the system achieved an overall classification accuracy of 97.9% (AUC > 0.95 across all subgroups), demonstrating the potential of AI-driven spectral pattern recognition in discriminating tumour-specific EV profiles (Chen et al. 2025). Collectively, these advances underscore how the convergence of AI with next-generation microfluidic technologies can dramatically enhance the efficiency and precision of EV isolation, offering new opportunities for rapid, reliable, and minimally invasive liquid biopsy in oncology (Figure 4A).

## 6. AI in the design of extracellular vesicle-based drug delivery systems

### 6.1 AI for functional characterisation of extracellular vesicles and prediction of carrier performance

From natural to engineered vesicles, EVs have undergone a paradigm shift from passive targeting vehicles to programmable delivery platforms. Native EVs inherently exhibit low immunogenicity and good biocompatibility, which confer natural advantages in drug delivery. Surface ligand modification and membrane biomimicry strategies further endow EVs with tissue-targeting capabilities (Frolova and Li 2022; Liu et al. 2023) (Figure 3F). For example, integrin β3, which is heterogeneously expressed across pancreatic cancer cells and stromal compartments, has been implicated in the homing of RGD-modified vesicles, thereby enhancing intratumoral paclitaxel delivery. In addition, RGD motifs are not specific to integrin β3 and can also interact with other integrins. Similarly, biomimetic vesicles based on hybrid membranes of cancer-associated fibroblasts (CAFs) and ovarian cancer cells (PH20/CCM) have been engineered to simultaneously target tumour cells and CAFs, enabling co-delivery of carboplatin and p65 siRNA, which enhances stromal penetration and immune infiltration (Al Faruque et al. 2022; Yao et al. 2025). Based on these developments, more sophisticated multimodal platforms have been developed. Engineered nanovesicles, for instance, can display an IL-21 fusion protein and a CD44-targeting peptide on their surface while co-encapsulating glucose oxidase and ferrocene, thereby combining starvation therapy, chemodynamic therapy, and immunotherapy to enhance antitumor efficacy (Li et al. 2025).

Despite these promising strategies, most approaches remain empirically driven and lack systematic frameworks for prediction and optimisation. Intrinsic EV heterogeneity, complex molecular cargo, and uncertain delivery pathways continue to pose major barriers to clinical translation (Loria et al. 2024). Against this backdrop, AI is increasingly being applied to support the development of EVs as more controllable therapeutic platforms, particularly through multiomics integration, nonlinear modelling, and high-dimensional data interpretation (Nam et al. 2024). For instance, in non-small-cell lung cancer (NSCLC), the application of an RF algorithm to EV-associated miRNA profiles led to the construction of a predictive feature panel comprising 45 miRNAs and three clinical variables (Zhang et al. 2022). This model successfully stratified patients into exceptional responders, resistant patients, and other categories, achieving sensitivities of 0.81–0.89 across multiple datasets, significantly outperforming PD-L1-based immunohistochemistry. These findings indicate that AI may support treatment-response prediction and patient stratification based on EV-associated molecular profiles and may thereby inform individualised treatment strategies.

AI has also been applied to identify candidate molecular factors involved in EV biogenesis and secretion. In the context of small RNA secretion, Zirak et al. developed ExoGRU, a GRU-based deep learning model for predicting the probability of small RNA secretion via EVs (Zirak et al. 2024). ExoGRU identified key RNA-binding proteins, including YBX1, HNRNPA2B1, and RBM24, as candidate regulators of small RNA secretion, while exoCLIP was used to map the extracellular RNA interactomes of HNRNPA2B1 and RBM24. These findings provide computational evidence for prioritising candidate regulators and sequence features in the design of EV carriers. More importantly, ExoGRU was extended to synthetic sequence design, enabling the prediction and optimisation of secretion potential. This approach successfully generated artificial small RNA sequences exhibiting high secretion capacity in vitro. Although these experiments provided support for some of the predictions, the associations identified by the model remain insufficient to establish causal regulation. The specific roles of individual RNA-binding proteins and sequence features in small RNA secretion therefore require further validation through targeted perturbation and functional experiments. Accordingly, AI-assisted prediction is currently better suited to screening and optimising candidate RNA sequences with high secretion potential, thereby narrowing the experimental search space and improving the focus of subsequent functional validation.

In the analysis of EV-associated phenotypes and prediction of therapeutic responses, researchers have developed a signal amplification strategy based on QNs combined with machine learning algorithms, enabling highly sensitive detection of EV-associated PD-L1. Using DNA-assisted assembly of QNs, this approach allows for dual-colour phenotypic profiling of circulating EV-associated PD-L1 and EpCAM, while machine learning algorithms analyse the detected fluorescence signals to distinguish cancer patients from healthy individuals and to predict responses to immunotherapy (Zhang et al. 2024). Moreover, incorporating additional features such as differences, products, and squared ratios of fluorescence signals further enhanced detection accuracy and predictive performance. These results indicate that AI can assist in interpreting complex EV-associated signals and predicting treatment response, with potential value for precision medicine applications. At present, AI applications in EV-based drug delivery mainly identify associations among EV molecular composition, secretion characteristics, and therapeutic response. Whether these associations are causal requires further confirmation through targeted perturbation, functional experiments, and independent experimental validation.

### 6.2 AI in structural optimisation and design of extracellular vesicle carriers

At the structural optimisation level, AI is increasingly being explored as a supportive and predictive tool to facilitate EV-based delivery systems. By leveraging multi-objective modelling and experimental design strategies, researchers can predict the relationships between EV physicochemical parameters and delivery performance, enabling coordinated optimisation of particle size, surface charge, protein density, and cargo loading modes (Raghav and Jeong 2021; Chen et al. 2024). Recently, large-scale machine learning driven analyses of nanomaterials have further accelerated the intelligent design of delivery systems. For instance, one study integrated data from 745 preclinical investigations to construct a multidimensional database encompassing nanoparticle physicochemical properties, biofunctionalization strategies, and in vivo performance. Using interpretable machine learning models, such as RF and SHAP analysis, the study identified key structural variables that critically influence tumour-suppressive efficacy (Mendes et al. 2024). Beyond bulk physicochemical parameters, AI-assisted analytical platforms have begun to reveal structural and compositional heterogeneity at higher resolution. Deep learning-based image and spectral analyses allow automated extraction of EV features such as size distribution, membrane integrity, and nanoscale heterogeneity, improving quality control and standardisation. In parallel, single-cell EV profiling has identified specific tumour subpopulations that preferentially release marker-enriched vesicles, such as HSP70- and EpCAM-positive EV, offering biological cues for the development of more selective targeting strategies (Song et al. 2022). Meanwhile, AI-guided protein structure prediction and molecular interaction simulations are being explored to inform membrane functionalization and surface engineering, with the aim of improving targeting specificity and stability. However, these applications largely remain assistive and exploratory rather than constituting direct structural redesign at the molecular level.

AI-assisted optimisation may also influence EV structural characteristics indirectly through biomanufacturing control. Because EV composition and membrane-associated functionality are intrinsically determined during biogenesis, modulation of production parameters can reshape vesicle molecular profiles (Lu et al. 2025). In this context, Bader et al. applied multi-objective batch Bayesian optimisation (MOBBO) coupled with a Gaussian process surrogate model to systematically screen culture conditions for mesenchymal stem cell-derived EV production. This machine learning-guided framework identified conditions associated with improved vesicle purity and functional activity, increasing the vesicle-to-protein ratio to approximately 6.2 × 10^9^ particles μg^−1^ and elevating surface-associated CD73 activity to nearly 30 U mg^−1^. These quantitatively measurable improvements illustrate the feasibility of AI-driven process optimisation as an indirect strategy for structure-informed functional enhancement (Bader et al. 2023). Overall, current AI applications in EV research predominantly focus on predictive modelling, characterisation, and process optimisation, whereas direct AI-enabled structural engineering of vesicle architecture remains relatively underexplored. Future advances in multimodal data integration, interpretable modelling, and experimental validation will be essential to translate AI from an assistive analytical tool into a more proactive design framework for clinically translatable EV delivery systems.

### 6.3 AI in modelling extracellular vesicle trafficking pathways and in vivo behaviour

EVs delivery involves a series of complex biological processes, including release, circulation, targeting, uptake, and intracellular trafficking, which exhibit pronounced spatiotemporal dynamics and tissue specificity (Zhao et al. 2024). Traditional approaches face limitations in resolution and throughput when monitoring these dynamic events, whereas AI-driven data modelling and image analysis offer transformative opportunities. For example, AI-integrated in vivo imaging techniques can be used to capture the organ-level biodistribution of EVs. Deep CNN trained on multitemporal tissue distribution images enable precise prediction of the organ-specific homing of EV in mice, including the liver, spleen, lung, and kidney. These models also provide insights into how specific EV modifications, such as membrane protein engineering or surface ligand conjugation, modulate their in vivo behaviour, offering a computational framework for rational design of EV-based delivery systems (Mi et al. 2024). This enables quantitative evaluation of delivery efficiency and rapid assessment of targeting specificity, thereby informing the rational design of EV-based therapeutics. Moreover, by developing machine learning models that integrate multimodal parameters, including EV size, surface charge, and membrane protein expression profiles, it is possible to predict both uptake mechanisms, such as macropinocytosis or receptor-mediated endocytosis, and affinities for specific cell types (Zhai et al. 2023; Jackson Cullison et al. 2024). Such integrative modelling promotes a bidirectional understanding of EV uptake efficiency and biodistribution patterns, thereby contributing to the optimisation of delivery outcomes and the generation of mechanistic hypotheses. At present, these AI models are primarily trained on murine biodistribution data derived from in vivo imaging, and their translatability to human physiology remains limited. Differences between mice and humans in hemodynamics, immune clearance, vascular barriers, organ physiology, and receptor expression may hinder the direct extrapolation of murine EV biodistribution and organ-homing patterns to humans. Further validation in human-relevant experimental systems and clinical datasets is therefore required before these models can reliably support clinical translation (Kim et al. 2026).

AI technologies have shown increasingly broad applicability in EV-based delivery systems. Conventional EVs often encounter challenges during systemic administration, as their biodistribution is governed by hemodynamic forces and biological barriers, which limit their ability to accurately reach tumour sites. The integration of AI algorithms enables researchers to dynamically predict EV migration trajectories in the bloodstream, based on large-scale simulations and experimental datasets, and to adjust ultrasound parameters in realtime to optimise the spatiotemporal distribution of drug release (Zhu et al. 2024; Shi et al. 2025). For instance, Akbar et al. highlighted the use of AI in microbubble-assisted EV delivery, where AI-driven optimisation of delivery pathways, coupled with acoustic signal analysis, allowed the establishment of a “signal-distribution-release” coupling model. This approach markedly improved delivery efficiency and targeting accuracy (Akbar et al. 2021). More importantly, the combination of AI with realtime imaging modalities, such as ultrasound and MRI, enables precise, dynamic monitoring of microbubble localisation, EV aggregation, and rupture, providing comprehensive insights into in vivo drug delivery processes and therapeutic performance (Akbar et al. 2021). Beyond oncology-focused studies, AI-driven delivery modelling has also been applied to non-tumour EV systems, such as lactic acid bacteria-derived EV, where AI-based target prediction and molecular docking were used to optimise cargo-target interactions and improve in vivo delivery efficiency, illustrating a broadly applicable paradigm for EV-based drug delivery optimisation (Wang et al. 2025) (Figure 4B). In the future, the combination of generative AI models with experimental feedback loops provides a comprehensive framework for the rational design of EVs. By systematically linking input parameters, such as EV size, surface charge, and functional modifications, to output outcomes including delivery efficiency, tissue distribution, and therapeutic efficacy, this approach enables a shift from static optimisation to dynamic, realtime regulation of EV systems. This closed-loop, multiscale modelling strategy integrates functional profiling, structural design, and system-level optimisation, offering a comprehensive platform to guide the development of next-generation intelligent EV-based therapeutic applications.

## 7. AI-enabled clinical translation and mechanistic hypothesis generation in extracellular vesicles

AI is increasingly being applied to mechanistic studies of EVs and to translational research efforts that bridge preclinical discovery and future clinical applications. On one hand, AI integrated with multiomics analyses can identify molecular signatures associated with EV-related biological processes and prioritise candidate pathways for further investigation. For example, differential gene expression analysis based on public datasets such as GEO and TCGA has revealed that EV-related genes are preferentially localised to specific cellular compartments and contribute to fundamental pathways, including metabolic regulation and cell cycle control (Zhu et al. 2022; He et al. 2025). In parallel, the integration of AI with high-throughput EV isolation technologies is accelerating the development of EV-based liquid biopsy, enabling noninvasive diagnostics across multiple cancer types (Yang et al. 2024; Liu et al. 2025) (Figure 4C). Zheng et al. developed an integrated pipeline that combined rapid sequential solid-phase extraction (SSEC) with MALDI-TOF MS for high-purity plasma EV isolation within two hours, followed by analysis of 220 clinical samples using an Exo-ANN neural network (Zheng et al. 2022). This system achieved 80% diagnostic accuracy (AUC 0.91) across breast cancer, pancreatic cancer, and healthy controls, demonstrating the potential of EV liquid biopsy approaches. Similarly, Song et al. developed a single-cell microfluidic platform with spatially barcoded antibodies to simultaneously profile five EV phenotypes and inflammatory factors across more than 1,000 cells (Song et al. 2022). Unsupervised clustering identified heterogeneous and highly secretory subpopulations in ovarian tumour cells, including HSP70^+^EpCAM^+^ EV-secreting cells, providing novel insights for studying intercellular communication. Notably, the primary role of AI is to identify associative patterns in high-dimensional data, narrow the range of candidate molecules and pathways, and characterise cellular subpopulations with specific functional or phenotypic features, thereby generating mechanistic hypotheses for subsequent experimental testing. However, true mechanistic elucidation, characterisation of biogenesis pathways, and validation of targeted therapeutic strategies still require complementary in vitro and in vivo functional experiments to define the mechanistic links among relevant EV components, signalling pathways, and cellular behaviours.

In a related study, Chen et al. combined DNA-PAINT with machine learning to quantitatively assess multiple EVs biomarkers in blood from cancer patients, achieving 100% classification accuracy in breast and pancreatic cancer samples (Chen et al. 2019), highlighting the potential of AI-assisted EV profiling to reduce confounding effects associated with bulk measurements and improve noninvasive cancer detection. For predicting therapeutic responses, Jia et al. integrated Raman spectroscopy with PCA, LDA, and SVM algorithms to develop an early-response prediction model for HER2^+^ breast cancer undergoing neoadjuvant therapy (AUC > 0.89), further enhancing accuracy to 94% using a HER2-specific capture system (Jia et al. 2025). Likewise, Qiu et al. developed the EV Latch isolation method and PURE-AID classification system for urine-derived EV, integrating markers such as PCA3, HOXC6, and DLX1 for prostate cancer screening (Qiu et al. 2025). In a cohort of 351 patients, PURE-AID achieved an AUC of 0.76, which increased to 0.80 when combined with PSA testing, reducing unnecessary biopsies by 54.3% while maintaining 86.8% sensitivity.

Despite these advances, several challenges remain for clinical translation. First, AI model performance heavily depends on specific datasets and isolation platforms, limiting generalisability across diverse tissue origins, pathological states, and individual variability (Kelly et al. 2019; Elguoshy et al. 2025). In clinical settings, EV phenotypes exhibit dynamic fluctuations in response to therapy, comorbidities, and metabolic state, and conventional static models may misclassify these variations as noise, potentially obscuring biologically relevant information. Second, most current models focus primarily on classification performance and lack sufficient causal interpretability to directly connect computational predictions with upstream molecular mechanisms, immune pathways, or cellular behaviours, which limits their clinical applicability (Couckuyt et al. 2022).

## 8. Discussion

EVs have emerged as important subjects of investigation in the field of precision oncology (Bode and Dong 2018; Del Bene et al. 2022). They participate in multiple pathological processes, including modulation of tumour immunity, promotion of angiogenesis, and establishment of the pre-metastatic niche (Chen et al. 2022). Beyond their biological roles in tumour progression, EVs also demonstrate potential clinical utility for early cancer screening, therapeutic response monitoring, and recurrence assessment (Tian et al. 2021; Zhou et al. 2021). In addition, their favourable biocompatibility and native cell membrane structure make them attractive candidates for drug delivery applications (Meng et al. 2020; Sharma et al. 2021). However, EV populations exhibit pronounced heterogeneity, which arises from inter-individual variability as well as differences in sample isolation and characterisation procedures (Witwer et al. 2013; Bordanaba-Florit et al. 2021). Current analytical and separation technologies often struggle to simultaneously achieve high throughput processing and precise discrimination of vesicle subpopulations (Li et al. 2025). Owing to its strengths in efficient data handling and complex pattern recognition, AI has therefore begun to be increasingly integrated into EV research.

Given the intrinsic complexity and multidimensional nature of EV characterisation data, single analytical approaches are often insufficient to achieve both accuracy and robustness in feature extraction and classification. Consequently, the development of integrative analytical strategies capable of incorporating multimodal and multisource information has become an important direction for advancing vesicle research (Zhang et al. 2025). AI, with its capacity for efficient processing and pattern recognition in large-scale and complex datasets, has demonstrated superior performance compared with conventional methods in fields such as medical imaging, genomics, and pathological analysis (Bi et al. 2019). In vesicle research, AI enables the integration of multimodal data, including Raman spectroscopy, interferometric scattering imaging, and proteomics (Bi et al. 2025), thereby improving the sensitivity of tumour-associated vesicle detection and enabling more refined characterisation of vesicle heterogeneity. In particular, for the interpretation of complex spectral signals such as surface-enhanced Raman scattering, deep neural networks exhibit strong feature learning capabilities and can establish extracellular vesicle classification models that reflect tumour-associated molecular alterations, ultimately enhancing the accuracy of tumour diagnosis and subtyping (Qian et al. 2022; Liu et al. 2025). However, the limited interpretability inherent to deep learning models remains a major barrier to their clinical translation and practical adoption. To improve model transparency and reliability, recent studies have introduced explainable algorithms, including methods such as LIME and SHAP, to visualise key decision-making features within the models. These approaches help increase clinicians’ understanding of AI-assisted extracellular vesicle-based tumour diagnostics and promote greater confidence in their clinical application.

Beyond its advantages in label-free spectral signal recognition, the integration of AI with high-throughput imaging platforms has further enhanced the characterisation of EVs morphology and molecular features. With the assistance of models such as CNN, automated systems can identify vesicle subtypes at the nanoscale with minimal human intervention, thereby reducing operator-dependent variability (del Real Mata et al. 2023). This capability is particularly advantageous in multichannel fluorescence or dark field imaging, where low-abundance subpopulations can be detected with improved sensitivity. The combination of automation and precision provides a technical foundation for efficient large-scale screening and classification of EVs (Bordanaba-Florit et al. 2021; Liu et al. 2025). Despite these advances, variations in image resolution, background noise, and cross-platform compatibility often lead to inconsistent model performance across different experimental settings. Moreover, the strong dependence of AI models on image quality may amplify technical biases introduced during data acquisition, potentially compromising reproducibility and comparability (Liang et al. 2022). To address these challenges, recent studies have proposed multimodal training strategies that integrate heterogeneous data sources to enhance model robustness and generalisability (Boehm et al. 2022; Dong et al. 2026). In addition, the incorporation of attention mechanisms enables models to focus more effectively on informative regions within vesicle images and spectra, reducing interference from background noise and improving the discrimination of subtle structural differences. Collectively, these methodological improvements are facilitating the transition of AI-based imaging models from laboratory analytical tools toward clinically translatable diagnostic support systems, thereby broadening their practical applications in extracellular vesicle research. Nevertheless, research on AI-assisted EVs analysis remains largely at the stage of methodological exploration and proof of concept, and model performance still depends substantially on experimental conditions and data quality. Factors such as image resolution, background noise levels, and differences among detection platforms may lead to variability in model performance across experimental settings (Huo et al. 2024). Recent efforts have attempted to mitigate these issues through multimodal training strategies and the incorporation of attention mechanisms, which enhance model adaptability to heterogeneous datasets and reduce interference from background noise during feature extraction (Liu et al. 2025).

Despite these technical advances, several systemic challenges continue to hinder clinical translation, including insufficient standardisation, dataset bias, and limited reproducibility. First, the lack of unified protocols for EV isolation, purification, and characterisation may introduce variability in data distributions, causing models to learn platform-specific or workflow-related technical signals rather than biologically meaningful features (Gandham et al. 2020; Bordanaba-Florit et al. 2021). Second, most current studies rely on single-centre, small-scale, or retrospective cohorts, which are prone to selection bias and increase the risk of overfitting. Consistent with observations in the broader field of medical AI, models that perform well during internal validation often exhibit reduced accuracy when evaluated in independent or ethnically diverse populations. For example, in a large multicenter study of an X-ray-assisted diagnostic system, model sensitivity declined markedly to 35.3 percent when applied to an asymptomatic screening population with a disease prevalence of only 2.2 percent (Kim et al. 2023). Similarly, in an independent validation cohort with greater racial and demographic diversity, the discriminative performance decreased from approximately 0.90 during internal testing to 0.85, with particularly pronounced reductions observed among Hispanic women and patients with a prior history of breast cancer (Hsu et al. 2022). Differences in data acquisition strategies, annotation protocols, and validation designs frequently result in inconsistent and difficult-to-reproduce performance across studies. Taken together, these findings highlight that the establishment of high-quality and standardised vesicle datasets represents a critical prerequisite for advancing the clinical translation of AI-driven extracellular vesicle research. To advance from proof-of-concept to clinical utility, the translation of AI-EV paradigms necessitates more than just performance metrics; it demands systemic support through standardisation, independent validation, and regulatory alignment.

At the level of clinical translation, real-world healthcare environments are inherently complex and nonstationary. Patient demographics, disease stage distributions, and therapeutic interventions continuously evolve, and the molecular composition of EVs likewise changes dynamically in response to disease progression and treatment exposure (Kumar et al. 2024; Greening et al. 2025). Such temporal variability presents additional challenges for AI-based EV analysis. At present, most AI-assisted EV studies still rely primarily on retrospective datasets, which may amplify selection bias and limit the ability of models to capture the full complexity of real clinical settings (Greening et al. 2025). The concept of the “AI chasm,” proposed by Kelly and colleagues, highlights that high accuracy achieved on retrospective data does not necessarily translate into meaningful clinical benefit in practice (Kelly et al. 2019). In this context, the performance degradation of models trained on static datasets in longitudinal follow-up or cross-institutional cohorts remains to be validated, highlighting an intrinsic limitation of current approaches (Cascarano et al. 2023). These considerations point toward important future directions for AI-enabled EV research. Incorporating dynamic modelling frameworks, continual learning strategies, and time-aware analytical approaches may improve model robustness and adaptability to evolving clinical environments, thereby enhancing their translational potential.

In future studies, the construction of multicenter databases and cross-platform data integration represents a key direction for the advancement of AI-enabled EV research. Increasing the diversity of sample sources can help mitigate dataset bias and improve model generalisability (Maleki et al. 2022). At the same time, issues related to data security and algorithmic ethics must be carefully addressed. EV-related datasets often contain sensitive patient information, and strict compliance with data sharing agreements and privacy protection regulations is essential during cross-institutional model development. From an algorithmic perspective, efforts should also be made to avoid bias toward specific sex, age, or ethnic groups, ensuring fairness and inclusiveness of AI systems in oncological applications (Chen et al. 2023). Importantly, AI should not be viewed solely as a tool for accelerating data analysis. Instead, it has the potential to be embedded throughout the entire EV research workflow, including optimisation of isolation strategies, automation of characterisation platforms, visualisation of vesicle heterogeneity, and guidance of functional validation studies (Bordanaba-Florit et al. 2021; Hendrix et al. 2023). The establishment of integrated intelligent systems spanning from sample processing to result interpretation may substantially facilitate the clinical implementation of AI-assisted precision medicine. Looking ahead, future development should shift from a narrow focus on algorithmic performance optimisation toward the construction of systematic translational pathways. On one hand, standardisation of sample handling procedures, data acquisition protocols, and quality control systems is required to establish interoperable cross-platform data frameworks. Leveraging large-scale, multicenter, and representative population cohorts will be critical for reducing dataset bias and enhancing model robustness and reproducibility at the source. On the other hand, prospective study designs and external validation are necessary to rigorously evaluate algorithm performance in real clinical settings. At the methodological level, multimodal data integration, continual learning strategies, and time-aware modelling approaches may further improve the ability of AI systems to capture dynamic biological processes. The deep integration of AI with EV research is therefore expected not only to provide more forward-looking strategies for precision cancer diagnosis and therapy but also to advance our understanding of intercellular communication and drive the development of next-generation biomedical engineering. With continued interdisciplinary collaboration and the establishment of high-quality data infrastructures, AI-based EV research is poised to achieve greater stability, interpretability, and translational relevance, ultimately supporting the development of next-generation personalised oncology strategies.

## 9. Conclusion

AI-driven multimodal data integration and liquid biopsy applications support EV research across isolation, characterisation, and biomarker discovery. AI algorithms applied to microfluidic platforms improve the purity and efficiency of EV isolation, and deep learning facilitates the integration of realtime imaging, Raman spectroscopy, and multiomics to enhance EV recognition and classification. Models based on machine learning show potential for multicancer early detection, dynamic therapeutic monitoring, and recurrence risk stratification. Data-driven design strategies can predict the biodistribution, uptake pathways, and delivery efficiency of EVs, supporting the transition to EV-based therapeutic platforms. With the continuous accumulation of multicenter, cross-platform EV datasets and the application of explainable AI and interpretability methods, reproducibility, generalisability, and clinical translation may improve. Collectively, these advances support precision oncology, enabling noninvasive diagnostics and strengthening EV-based drug delivery.

## Data Availability

The data that support the findings of this study are available from the corresponding author upon reasonable request.
